# Anthocyanin Colorimetric Strip for Volatile Amine Determination

**DOI:** 10.1155/2020/1672851

**Published:** 2020-06-17

**Authors:** Ricarl Irish F. Agunos, Danilet Vi M. Mendoza, Michael Angelo S. Rivera

**Affiliations:** ^1^Chemistry and Environmental Science Department, College of Arts and Sciences, Nueva Ecija University of Science and Technology, Cabanatuan City 3100, Philippines; ^2^Center for Environmental Research, College of Arts and Sciences, Nueva Ecija University of Science and Technology, Cabanatuan City 3100, Philippines

## Abstract

Food freshness is one of the main concerns of consumers. Food spoilage is mainly caused by contamination and microbial growth in which the latter produces volatile amines in the process. Several methods have been used to determine volatile amines to indicate food freshness, and indicator films are deemed as the most time-efficient and economical. In this study, anthocyanin was extracted from mangosteen rind as a natural dye indicator and was incorporated in a chitosan/PVA polymer matrix. The film with different concentrations of anthocyanin extract (5%, 15%, and 25%) was prepared and tested for their sensitivity to 136 ppm ammonia vapor followed by colorimetric analysis using ImageJ software. The film with 25% anthocyanin yielded the most visible color change upon exposure to ammonia vapor. The color changed from pink to yellowish-brown within 14 minutes of exposure. The RGB-converted images of the film with 25% anthocyanin extract showed gradual loss of red coloration being replaced by cyan spots. FTIR spectra showed incorporation of anthocyanin to the chitosan/PVA matrix with the decrease in the intensity of the C-N stretching peak. Thermogravimetric analysis showed that the film has high thermal stability with onset temperature of 310.43°C. Thus, the film developed is an excellent candidate for optimization and production of a thermally stable amine detector for food products.

## 1. Introduction

Food safety is important for consumers for health and economic reasons. Foodborne diseases brought about significant burden yearly. These diseases are deadly if not given enough attention especially to children below 5 years of age. These cause about 33 million deaths yearly worldwide and about 50,000 deaths in the Southeast Asia region alone [1].

Meat products, like pork, are highly susceptible to contamination and spoilage caused by microbial growth [2, 3]. Although freezing can inhibit the activities of microorganisms and enzymes, spoilage still occurs during the manufacture, transportation, and long-time storage processes. Therefore, in order to ensure food safety, it is important to monitor the freshness and quality of pork during storage in a real-time manner [4].

Traditional quality indices (total aerobic bacterial counts, pH, and total volatile basic nitrogen) for pork freshness had been studied to be related to biogenic amines. As the pork freshness decreases, biogenic amines increase [5]. The formation of biogenic amines (BAs) in food results from the enzymatic amino acid decarboxylation due to microbial enzymes and tissue activity [6]. This microgradient production of BAs results to an increase in total volatile basic nitrogen (TVB-N) concentration in meat products [7]. Thus, it increases the pH of the growth medium. Recently, this is also determined to be related to the rampant resistance of microorganisms to antibiotics. Aerial exposure to the bacterial volatile amine compound modifies antibiotic resistance of physically separated bacteria by raising culture medium pH [8]. Thus, this can be used to monitor the freshness and safety of food.

Anthocyanins are water-soluble pigments most commonly present in flowers, fruits, and vegetables. They are responsible for the red, blue, and purple coloration of plants [9]. Chemical structures of anthocyanins differ depending on the pH of the solution. Thus, the use of anthocyanin in monitoring pH conditions can be an advantage due to the release of different colors at various pH.

The study is focused on the development of a chitosan/PVA-based colorimetric strip with anthocyanin extracted from mangosteen rind as a volatile amine indicator.

Specifically, the study is aimed at preparing a colorimetric film by incorporating the anthocyanin extract (5%, 15%, and 25%) into the chitosan/PVA matrix, testing the sensitivity of the prepared film to volatile amine depending on time of exposure, determining the colorimetric changes in the film using ImageJ software, and characterizing the developed film strip.

## 2. Materials and Methods

### 2.1. Sample Collection and Preparation

Mangosteen fruits were purchased from the local market of Cabanatuan City, Nueva Ecija. The rinds were manually separated from the pulp, cut, and crushed in a blender before being stored at 7°C until use.

### 2.2. Extraction of Anthocyanin

Mangosteen extract was prepared according to the method used by Silva-Pereira et al. [10] with slight modification. To 150.0 g of mangosteen, 160 mL of ethanol-water (7 : 3) was added. HCl (1 mol/L) was also added to adjust the pH of the sample to 2.0. The resulting mixture was macerated for 24 h and protected from light. After maceration, it was filtered, and the extract was centrifuged at 2000 rpm for 10 minutes using a Spectrafuge 6C centrifuge with 15 mL capacity and maximum speed of 6500 rpm at 120 V. The resulting supernatant was then filtered on Whatman paper no. 1. The filtrate was evaporated using a rotary evaporator at 40°C for one hour and then stored in the dark at 4°C until use.

### 2.3. Confirmatory Tests for Anthocyanin

The confirmatory tests for anthocyanin were performed using standard procedures.

*Sulfuric acid test*: 1 mL of concentrated H_2_SO_4_ was added to 2 mL extract. The presence of anthocyanin would be indicated by orange coloration of the interface (Dipjyoti et al., 2016).

*Sodium hydroxide test*: 2 drops of 1 N NaOH were added to 2 mL of extract. The presence of anthocyanin would be indicated by blue to bluish-green coloration (Vijisaral et al., 2013).

### 2.4. Preparation of Anthocyanin/Chitosan/PVA Film

PVA was prepared by dissolving 10 g of polymer powder in 100 mL of distilled water under stirring at 70°C until complete dissolution occurred. Chitosan was prepared by dissolving 1 g of chitosan powder in 100 mL of aqueous acetic acid (1 mL/100 mL) under stirring at room temperature until it was completely dissolved. The chitosan/PVA mixture was prepared with a ratio of 7 : 3 (*v*/*v*), and the anthocyanin extract was added in different concentrations (5%, 15%, and 25% of the total volume of the mixture). A 1.5% solution of sodium tripolyphosphate (Na_5_P_3_O_10_) 0.1% (*w*/*v*) relative to the total volume of the mixture was also added to promote cross-linking. The films were prepared by casting 15 mL of the mixture in a 90 mm Petri dish, and then, the plates were placed in moderate temperature (35°C) for 72 h to avoid degradation of dye while removing the remaining solvent. The resulting films were peeled off the plates and were kept in sealed bags at 4°C in the dark until use [10, 11].

### 2.5. Sensitivity of the Film to Volatile Amine

Determination of the sensitivity of the films to volatile amines was carried out utilizing the method used by Zhai (2017). The colorimetric films were cut into 10 × 10 mm squares and hang up in a 500 mL Erlenmeyer flask, 1 cm above the ammonia solution (80 mL, 8 mM) at 30°C for 20 minutes. The flask was placed in a lightbox, and the films were monitored every two minutes. The films were also tested in water vapor as the control.

### 2.6. Colorimetric Response Determination

The color response of the films upon exposure to volatile amines was investigated using digital image analysis software, ImageJ. The films were photographed in a lightbox, and the images were analyzed using the software. Absorbance values of the films were obtained from the digital images by measuring the RGB (red, green, and blue) values [12, 13].

### 2.7. Characterization of the Film Strip

The chitosan/PVA control film and the colorimetric film with the highest sensitivity with ammonia were subjected to FT-IR spectroscopy using a SHIMADZU IRPrestige-21 spectrophotometer to analyze and to verify the chemical interactions between the film components [10].

The film strip's thermal stability was measured using a TGA analyzer (Perkin Elmer TGA 4000). The heating rate was 10°C per minute and 25-500°C temperature range [14].

## 3. Results and Discussion

### 3.1. Extraction of Anthocyanin

**Figure 1:**
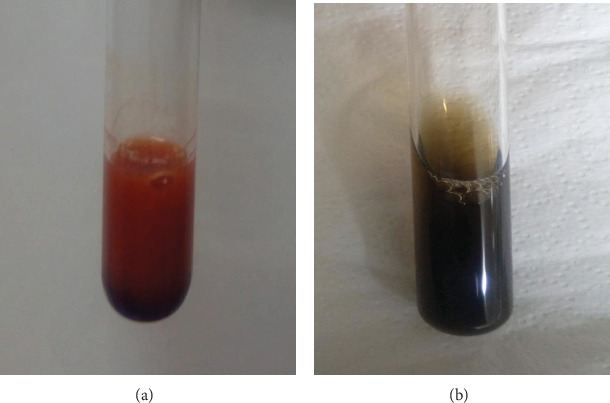
Confirmatory tests conducted for anthocyanin extract showing reddish-orange solution upon addition of H_2_SO_4_ (a) and yellowish-brown solution in the presence of 1 N NaOH (b).

Anthocyanin was extracted from the mangosteen rind using acidic ethanol. Bright red extract was obtained. The presence of anthocyanin was confirmed chemically due to the development of reddish-orange color of the solution upon addition of H_2_SO_4_ (Figure 1). Moreover, dark bluish-green solution was also observed upon addition of 1 N NaOH which quickly changed into yellowish-brown.

Confirmatory tests for anthocyanins involve acidic and basic solutions since forms of anthocyanin are pH dependent. Anthocyanins undergo molecular transformations when reacting with acids and bases. Generally, in an acidic medium, the predominant form is that of the flavylium ion, which gives a red color. At slightly higher pH (2-4), the anthocyanin forms anhydrobase anion which gives bluish to bluish-green coloration [15]. While at basic pH, it forms a light yellow solution due to the predominance of the chalcone form.

Slight variations in these expected color changes were observed since the color change may vary slightly depending on the type of anthocyanin and other factors like temperature and stability [16]. As based on the study of Chaovanalikit et al. [17], anthocyanins present in mangosteen rind are cyanidin-3-sophoroside and cyanidin-3-glucoside which are responsible for its red pigment.

### 3.2. Preparation of Colorimetric Film

**Figure 2:**
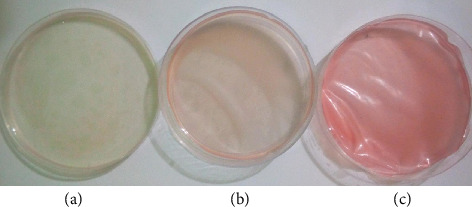
Developed colorimetric films with 5% (a), 15% (b), and 25% (c) anthocyanin extract casted on a Petri dish.

Colorimetric films were developed by casting the chitosan/PVA mixed with 5%, 15%, and 25% of the anthocyanin extract on a 90 mm Petri dish. The films with 5% and 15% anthocyanin extracts were light yellow and peach in color, respectively, while the film with 25% anthocyanin extract was pinkish-red (Figure 2). It is evident that the color of the film is dependent on the percentage of the anthocyanin extract incorporated on the polymer matrix.

### 3.3. Sensitivity of the Prepared Film to Volatile Amine

**Figure 3:**
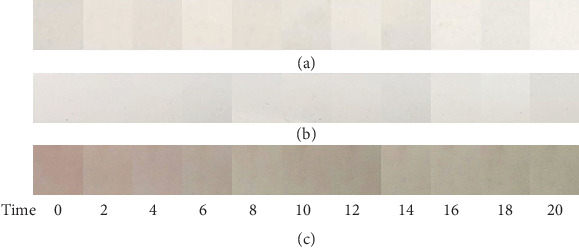
Color changes of the films with (a) 5%, (b) 15%, and (c) 25% anthocyanin extract per exposure time in minutes to ammonia vapor.

The films were tested for its color change response towards basic gas. The films were observed for 20 minutes, and photographs were taken every 2 minutes.  Figure 3 shows the response of the films to ammonia vapor. After being exposed to the ammonia vapor for 20 minutes, the film with 25% anthocyanin extract showed visible color change from pinkish-red to pale yellow. This film contained the most dye compared with the other two treatments making it the most tinted film. This most tinted film resulted to the most evident color changes using the naked eye. According to Zhai (2017), the mechanism for the colorimetric changes of the films was that the volatile NH_3_ firstly combined with H_2_O contained in the colorimetric film to form NH_3_·H_2_O which then hydrolyzed to produce NH_4_^+^ and OH^−^, the latter of which induced the color change of the films due to increase in pH.

**Figure 4:**
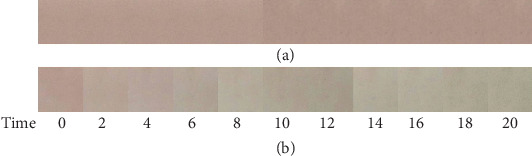
Color changes of the film with 25% anthocyanin extract per time of exposure in minutes with (a) water vapor and (b) ammonia vapor.

Another time study was conducted where the film containing 25% anthocyanin extract was exposed to water vapor and ammonia vapor. This was done in order to determine whether water vapor which is usually present in moist food products can interfere with the color change of the film which can cause false-positive results. It was observed that the film exposed with water vapor did not show any color change proving that the color changes in the colorimetric film was caused only by its interaction with ammonia vapor as shown in Figure 4.

### 3.4. Colorimetric Analysis of the Prepared Film

The colorimetric changes of the film upon exposure to the volatile amine were observed. The photographs of the films were processed using ImageJ, and RGB values were measured. Color changes from pink to yellowish-brown were visible in the actual photographs of the film with 25% anthocyanin extract. However, the calculated mean RGB values which are used to do the color gradient showed the film turning gray. This is due to the shadow casted by the Erlenmeyer flask inside the lightbox. For that reason, the images were further processed in the software by subtracting its background and threshold using the RGB values resulting to RGB-converted images.

**Figure 5:**
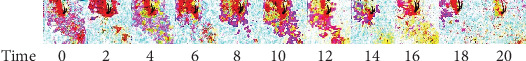
Colorimetric changes of the RGB-converted images of the film with 25% anthocyanin extract per time of exposure in minutes to ammonia vapor.

Figure 5 shows how the film loses its red and magenta coloration the longer it was exposed to ammonia. The film was also observed to have cyan as the most dominant color as time of exposure increases. Magenta and cyan resulted from the addition of red-blue and green-blue, respectively. The colorimetric changes of the film from more red and magenta to more cyan spots showed that it became dominated by green and blue which can be observed after 14 minutes of exposure.

### 3.5. Characterization of the Colorimetric Film

**Figure 6:**
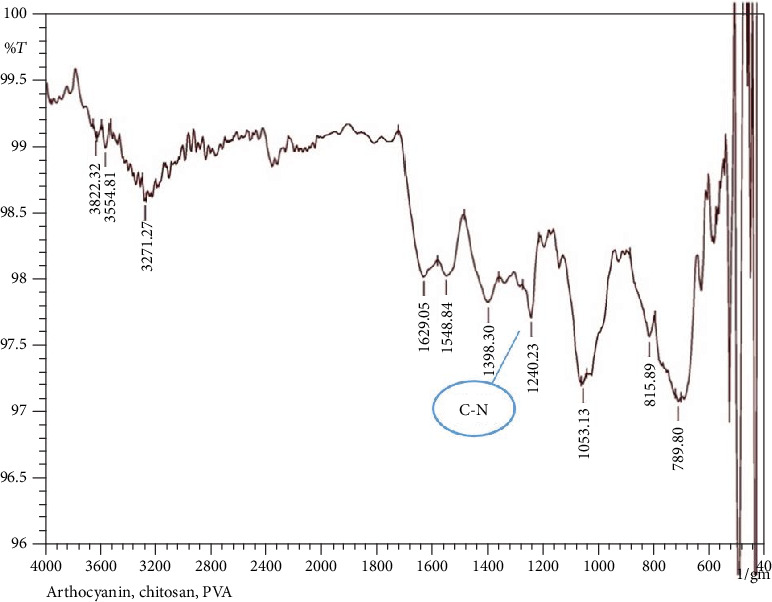
FTIR spectra of the developed anthocyanin colorimetric film showing the smaller peak of the C-N stretching at around 1240 cm^−1^ in comparison to the spectra of the control.

**Figure 7:**
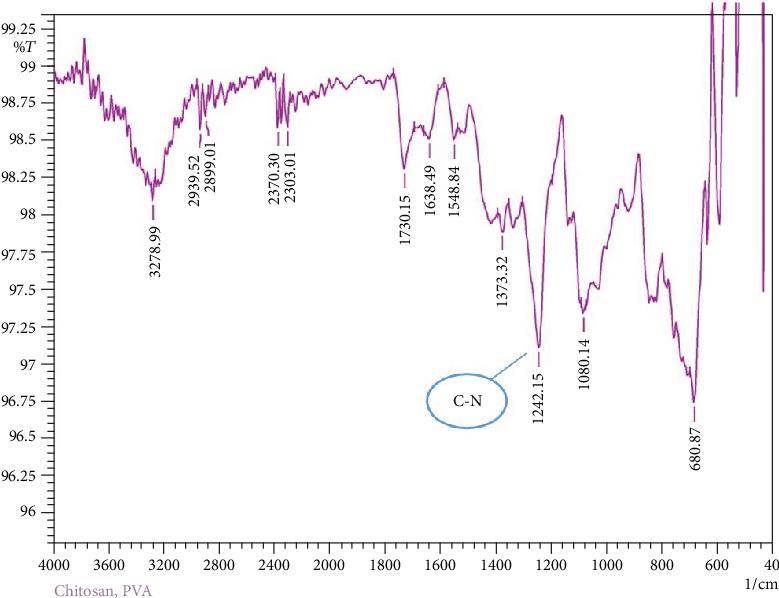
FTIR spectra of chitosan/PVA control film showing the characteristic C-N stretching peak of the chitosan polymer chain at around 1240 cm^−1^.

FTIR was used to observe and compare the changes in the functional groups of the chitosan/PVA film upon addition of anthocyanin extract. The colorimetric film absorption spectra (Figure 6) showed peak at 1240 cm^−1^ (C-N stretching) which is assigned to the chitosan polymer chain. The peak had a decrease in intensity compared to the control film spectra (Figure 7) due to the interaction of the carbonyl group of the anthocyanin to the chitosan polymer. This suggests that the chitosan polymer has hydrophobic interaction with the anthocyanin extract similar to the result of the study of Tan et al. [18, 19] where this interaction leads to the formation of the chitosan anthocyanin complex.

**Figure 8:**
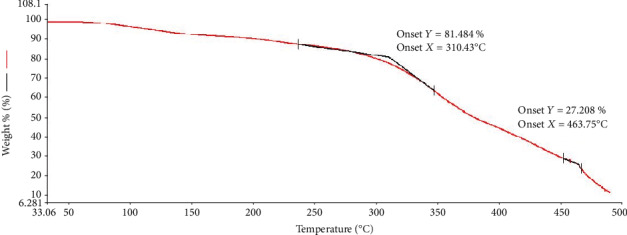
TGA plot of anthocyanin colorimetric film showing the onset temperature at 310.43°C.

The limit of temperature of the film was also determined. The TGA plot (Figure 8) showed slight weight loss (approximately 8%) at 100°C due to the loss of moisture from the film. The film was still stable until it reaches approximately 240°C, and the onset temperature was 310.43°C which denotes at which the weight loss began. This onset temperature is slightly lower as compared to that of pure chitosan/PVA film which is around 340°C based on the previous study of Hassiba et al. [20]. Anthocyanins usually have stable color at lower temperature of 60°C [21]. The result suggests that incorporation of the anthocyanin to the polymer matrix increased its thermal stability including its color. Lastly, the film started undergoing degradation at 463.75°C. Thus, the developed colorimetric film is a great candidate as raw material in producing a thermally stable indicator for food spoilage thru its interaction with volatile amine or change in pH.

## 4. Conclusions

A colorimetric film was developed in this study by incorporating the anthocyanin extracted from mangosteen rind to the chitosan/PVA matrix. A tinted film was produced using 25% of the extract. The film was determined to be sensitive to 136 ppm of ammonia vapor upon exposure at 14 minutes which caused a color change from reddish pink to light yellow due to the increase in pH. The color change was mainly due to the dominance of cyan spots in the film as observed using ImageJ software. FTIR analysis of the film suggests the formation of the chitosan anthocyanin complex, and TGA results showed the onset temperature of 310.43°C and start of degradation at 463.75°C.

These results suggest that the developed film is a great candidate as raw material in producing a thermally stable indicator for food spoilage thru its interaction with volatile amine or increase in pH.

## Data Availability

For data underlying the findings of the study, researchers may send an email to the authors.
